# Transforming language research from classic desktops to virtual environments

**DOI:** 10.1038/s41598-025-08319-1

**Published:** 2025-07-02

**Authors:** Francisco Rocabado, Laís Muntini, Omar Fahmi Jubran, Thomas Lachmann, Jon Andoni Duñabeitia

**Affiliations:** 1 Centro de Investigación Nebrija en Cognición, Universidad Nebrija, Madrid, 28043 Spain; 2 Center for Cognitive Science, RPTU Kaiserslautern, 67663 Kaiserslautern, Germany

**Keywords:** Psychology, Human behaviour

## Abstract

Virtual Reality (VR) offers novel opportunities for investigating human perception beyond conventional laboratory settings, facilitating the study of naturalistic behavior with controlled virtual environments. To benefit from this technology, the foundational aspects must be compared to traditional personal computer (PC) monitor setups. The validity and reliability of stimuli presentation and response collection must be established to ensure that any findings can be attributed to experimental variables, not the method. To address this, we designed a single-word recognition (lexical decision) task administered in both VR and PC monitor setups. Stimulus presentation was controlled across tasks, visual angles were matched, and responses were gathered via VR controllers in both settings. Results replicated the lexicality effect (i.e., faster word than pseudoword reading) in both setups. Reaction times and error rates showed no significant differences between VR and PC monitor setups, underscoring VR’s utility for collecting reliable behavioral data in language studies. These results demonstrate that VR-derived mental chronometry measures yield findings comparable to conventional methods, establishing a benchmark for future immersive research.

## Introduction

Virtual reality (VR) technology enables the creation of lifelike 3D environments where users can interact with a “virtually real” world^1,2^. Over recent years, advances in software and hardware have addressed many of its initial technical limitations, such as resolution and ergonomic issues. These developments increased the degree of immersion, i.e., the user’s sense of “being” in the virtual environment^3,4^and VR technology became more affordable and increasingly viable for psychological and linguistic experiments^5–9^.

Building on these technological advances, VR has opened new experimental possibilities across cognitive psychology and psycholinguistics. In cognitive psychology, VR has facilitated the study of human perception and action (e.g^8,10–12^. , executive functioning (e.g^13^. , and memory (e.g^14^. , ; see^15^with enhanced experimental control across participants. In the field of psycholinguistic research, VR was used to replicate established paradigms, such as the visual-world paradigm^16,17^language cued-switching tasks^18^and language comprehension tasks^19^. Some studies have leveraged VR to conduct experiments that are difficult to perform in the real world^8,20–22^or to recreate specific communicative dynamics from the real world^23,24^. However, while VR has been increasingly used to study sentence- or discourse-level language comprehension, much less attention has been given to the processing of individual lexical items, i.e., single words^25^. In addition, foundational aspects of VR research, such as stimulus presentation and response collection, and their comparison to traditional Personal Computer (PC) monitor setups remain underexplored^10^.

Single-word recognition and processing are key interest areas in psycholinguistic research (see^26^ for a review) because they are the primary interface through which the act of reading occurs^27^. Words are well-defined minimal lexical units, with characteristics such as orthography, phonology, semantics, and syntax, that are processed in either automatic or attention-driven processes^28^. Thus, numerous behavioral and neuroimaging studies have used single words to explore these processes^29^yet nearly all have relied on traditional laboratory setups. Considering that a significant portion of the written information encountered in daily life predominantly relies on single words^30^e.g., on billboards or street signs, VR enables the study of dynamic scenarios in which single words appear.

As a first step toward popularizing VR as a methodological tool for single-word processing research, Mirault and colleagues conducted a series of experiments in VR. They explored if this technology could be implemented to facilitate testing children in noisy environments without requiring external assessors or equipment (i.e. eye-tracking device;^31,32^. To assess children’s lexical access, they used a lexical decision task (LDT)^31^where participants are instructed to identify a variety of letter strings as either words or pseudowords (e.g., FLIRP), as fast and as accurately as possible (see^33^ for an overview). They illustrated how VR could offer precise stimulus presentation and attentional control within a neutral environment, correlating reading performance in VR to classic fluency measures^31,32^. Nevertheless, more research is needed to establish the comparability between performance in VR and conventional PC monitor setups using VR controllers.

The present study reproduces the LDT within a plausible real-life context, a virtual classroom, directly comparing performance metrics, i.e., Reaction Times (RT) and Accuracy, obtained in two task setups, PC monitor and VR, using a Head Mounted Display (HMD). Visual angles, task design, and response methods were matched across setups, with responses collected via VR wireless controllers. The study aims to replicate the lexicality effect (i.e., faster responses for words than pseudowords) in VR, and investigate whether the results are comparable across setups, in line with previous experiments^10,34^. Demonstrating such equivalence ensures that any future behavioral differences observed in more complex or naturalistic VR environments can be confidently attributed to experimental variables rather than technical discrepancies.

## Methods

### Participants

The final sample consisted of a total of forty Spanish male undergraduate students and employees with university education from Nebrija University who participated in the present study in exchange for economic compensation. Age ranges were 18–59 (*M* = 26.8, SD = 9.28). All participants had normal or corrected-to-normal vision, showed no cognitive impairments as per their results in the Cognitive Assessment Battery (CAB) PRO (CogniFit Inc., San Francisco, CA), and were unaware of the experiment’s purpose. Before the experiment began, they were asked to read and sign an informed consent form for data collection. G*Power^35^ was used to estimate the sample size needed to replicate a large effect size (F = 0.25; 1-β = 0.95), at a significance criterion of α = 0.05, for a study of one group and 4 measures (two stimuli type; word and no-word, and two task setup; PC and VR) as in Jubran et al.^10^. Given the result of this analysis, it was identified that at least 36 participants would be needed before the research was conducted, it received full ethical approval from the Research Ethics Committee at Universidad Antonio de Nebrija (UNNE-2022-0017). The experiment was performed following all applicable rules and ethical guidelines, including the Declaration of Helsinki, which protects the rights and well-being of research participants.

### Materials

One hundred five-letter Spanish words were selected from SPALEX, the Spanish lexical decision database^36^. All words received 100% accuracy scores in the study by Aguasvivas et al. The mean Zipf frequency based on the EsPal corpus^37^ was 4.7 (range 4.1–6.3). Additionally, one hundred, five-letter word-like pseudowords were also selected from the same database. The mean accuracy of the responses to these pseudowords in the Aguasvivas et al. study was 100%. The accuracy referenced here pertains to participant performance in the SPALEX lexical decision task, from which these words and pseudowords were sourced. The Zipf frequency metric reflects the log-scaled frequency of a word in a given corpus, offering a more interpretable and normalized frequency index^38^with higher values indicating more commonly used words.

### Virtual reality materials

**Fig. 1:**
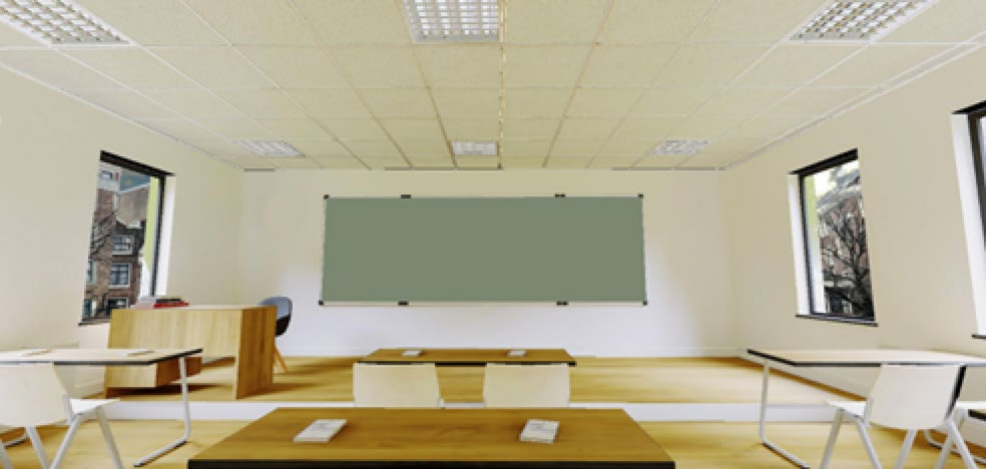
View of the main model used in the experiment, acquired from Sketchfab repository^39^.

The experimental scenario was presented in a VR setup using an HMD. A 3D building model was acquired from the 3D model repository Sketchfab^39^ and used as the main scenario, consisting of a realistic classroom that included chairs and desks, and a main blackboard to be used as a presentation canvas. Edits were made using Blender^40^ to adjust lighting and an HDRi background was added for increased immersion (see Fig. 1).

### Apparatus

The VR task was administered through the Vizard 6 programming platform, which operates on a Python 2.7-based system^41^. The experiment was conducted on a high-performance gaming laptop computer, featuring an Intel Core i7-10750 H processor (2.6 GHz), a Windows 10 operating system (64-bit), 32 GB of RAM, and an NVIDIA GeForce RTX 2070 graphics card to ensure a high-quality presentation. To ensure optimal device performance and communication between the computer and the HMD, the battery-saving settings were disabled throughout the experiment.

The HTC VIVE Pro HMD, with a resolution of 2880 × 1600 pixels and a field of view of 110º, was used to present the VR environment and stimuli to the participants. Although eye-tracking data were not collected, the eye-tracking calibration procedure involving a 5-point fixation method was executed successfully for all participants to ensure proper headset placement and eye-to-eye adjustment. The Motion Smoothing system in SteamVR was disabled to maintain a constant refresh rate throughout the experiment. The participants’ viewpoint within the VR environment was fixed but permitted a 360-degree rotation around their axis. For the 2D presentation, we relied on a 20” LCD DELL monitor at a full HD resolution 1900 × 1080 resolution.

Task setup and procedure were designed to minimize cybersickness^42,43^. The task did not include any moving objects or rapid changes in scenery. Participants performed the task while seated, and only responded by using buttons on the VR controllers. Furthermore, participants were instructed to report any discomfort, disorientation, or nausea, during, and after the task. They could stop the experiment at any time without explicit reason. The experiment duration was controlled, and all participants completed the task without reporting negative symptoms. Informal debriefing confirmed that the experimental setup did not induce any noticeable physical discomfort.

### Task and procedure

Participants completed two versions of a lexical decision task across two setups: one in VR and one on PC. In each trial, they decided whether a visually presented letter string was a real Spanish word or a pseudoword by using the trigger button of the right or left VR controller. Participants were instructed to respond as quickly and accurately as possible. Each setup consisted of 200 trials (100 words and 100 pseudowords), presented in random order. The order of setups (VR first vs. PC first) was counterbalanced across participants. A 500 ms inter-trial interval and a 3000 ms response window were implemented to standardize task pacing.

The same set of stimuli was used across both setups to allow for a direct within-subject comparison between VR and PC setups. Given the short exposure time per item and the temporal separation between setups, potential familiarity or repetition effects were minimized and evenly distributed across them.

Participants responded using the VR headset controllers in both setups by pulling the trigger located on the back of the right- or left-hand controller. Although participants were not explicitly instructed on how to hold the controllers, the trigger placement naturally encouraged the use of the index finger, ensuring a consistent response method across all trials.

**Fig. 2:**
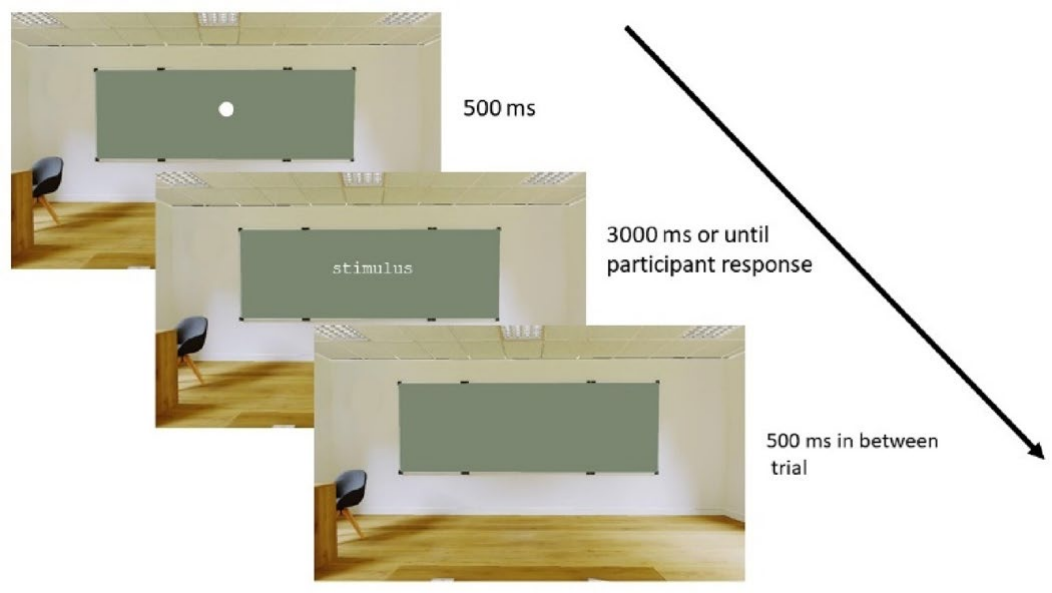
Trial structure of the lexical decision task.

All stimuli were presented in white (hex-code: #FFFFFF) Courier monospaced font (12pt) on a grayish-green background (hex-code: #7b8771), ensuring uniformity across setups. A 500 ms blank screen interval separated each trial (see Fig. 2). This design ensured consistent visual presentation and response collection across both setups.

To ensure perceptual consistency across setups, we matched the visual angle of the word stimuli between the VR and PC setups. This was achieved by calculating the subtended angle based on the participant’s viewing distance to the stimulus plane. In the VR setup, we used the Vizard Inspector tool to measure the virtual distance from the user’s viewpoint to the blackboard and adjusted the stimulus size accordingly to produce a horizontal angle of approximately 4.6°. In the PC setup, we replicated this angle by adjusting the font size and screen distance to match the perceived dimensions. These adjustments were validated through automated inspection routines embedded in the experiment code, ensuring consistency in the visual experience across both setups.

### Data manipulation and analysis procedure

Reaction time (RT) and accuracy data were preprocessed using R^44^ and RStudio^45^. Three participants were excluded due to technical issues, resulting in a final sample of 37 participants (age range = 18–59 years; M = 27.1, SD = 9.59).

The total number of trials (*N* = 14,800) was equally allocated between the two experimental setups (*N* = 7,400 for PC and *N* = 7,400 for VR). Next, a standard deviation (SD)-based trimming procedure was applied, excluding RTs exceeding ± 2.5 SDs from each participant’s setup-specific mean^46,47^. A total of 223 trials (3.11%) were removed from the PC setup and 202 trials (2.80%) from the VR setup. Most exclusions reflected upper-bound outliers, with only one lower-bound outlier detected in the PC setup.

**Fig. 3:**
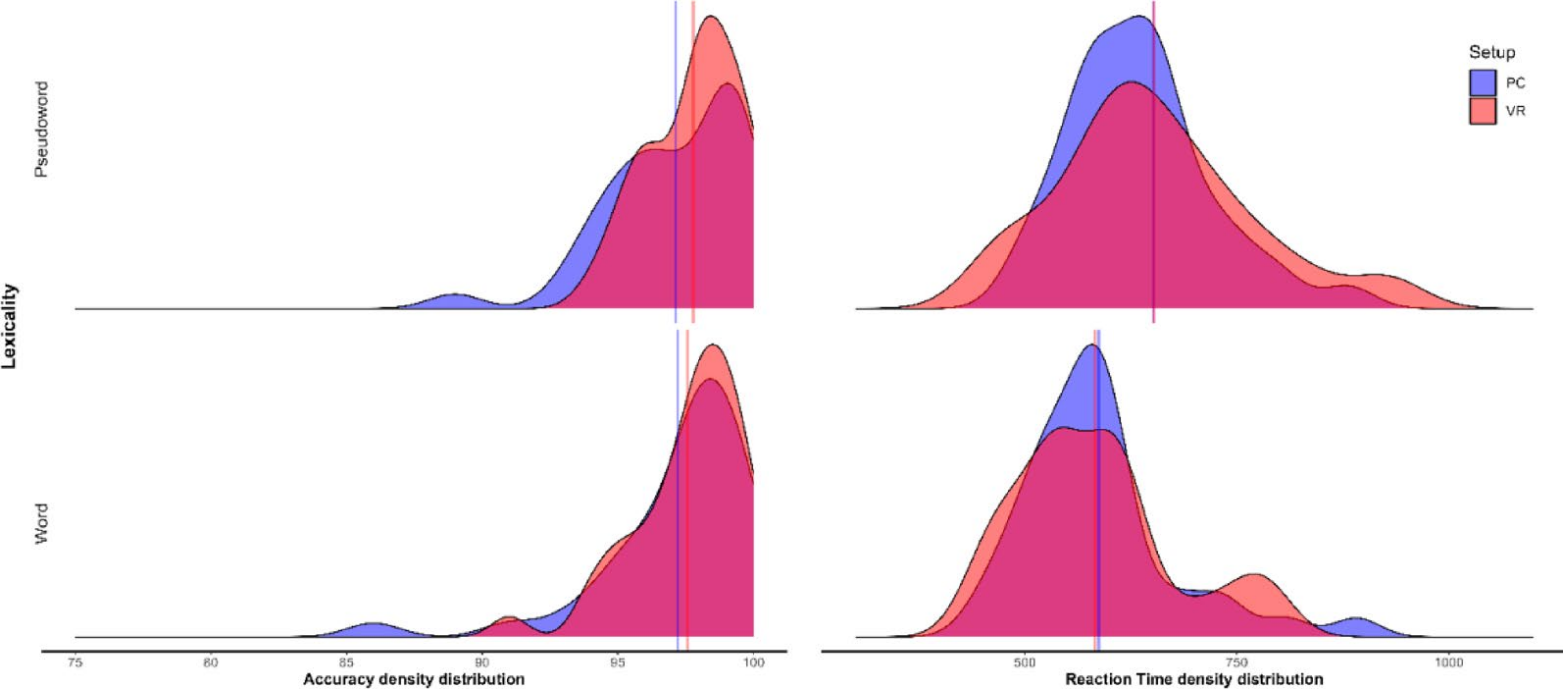
Accuracy percentages and RT (ms) density distribution across Lexicality levels. Red and blue lines represent the means in each setup.

Trials with no response or incorrect responses were excluded before RT analyses (2.97% from the PC; 2.41% from the VR), yielding 7,181 and 7,222 valid trials for the PC and VR setups, respectively. Only results based on trimmed data are reported. Descriptive statistics (means and standard deviations) and distributions for RT and accuracy were computed for each Setup and Lexicality level to provide a general overview of performance patterns (see Table 1; Fig. 3).

For inferential analyses, a repeated measures ANOVA with a 2 × 2 within-subjects design was conducted to examine the effects of Lexicality (word, pseudoword) and Setup (PC, VR) on RT and accuracy. Mean RTs were computed at the trial level using only correct responses.

To complement the preliminary analyses and assess the robustness of the results across participants and items, Linear Mixed-Effects Models (LMMs) were fitted following similar procedures to those outlined by Huettig et al.^17^using the lme4 package^48^ and lmerTest package^49^ in R (version 4.5.0). Three progressively simpler models were evaluated. The maximal model included Lexicality × Setup interaction as a fixed effect, with random intercepts and random slopes for Lexicality by participant and by item. This model failed to converge (degenerate Hessian matrix with negative eigenvalues), suggesting overparameterization.

A second model, which removed the interaction from the random structure while retaining random slopes, converged but resulted in a singular fit, indicating redundant parameters. A final simplified model, including only random intercepts for participants and items, achieved satisfactory convergence. All models were estimated using restricted maximum likelihood (REML) with the bobyqa optimizer (maxfun = 200,000) and Satterthwaite’s approximation for degrees of freedom. Diagnostic plots (residual vs. fitted, Q-Q, and density plots) indicated that model assumptions were reasonably met, although minor deviations were observed in the upper tail of the Q-Q plot.

Finally, to further account for variability related to age, an additional model included Age as a random grouping factor, allowing random slopes for the Lexicality × Setup interaction by age, following recommendations by Brauer and Curtin^50^. However, this model failed to converge (non-positive definite Hessian matrix), likely due to minimal age-related variance or overparameterization of the random effects structure.

**Table 1:** Mean reaction times and accuracy (percentage of hits) across lexicality conditions and setups. Standard deviations are provided in parentheses.

	Reaction Times (ms)		Accuracy	
Setup	Word	Pseudo-word	Word	Pseudo-word
PC (monitor)	587 (92)	652 (130)	97.2 (2.80)	97.1 (2.46)
VR (HMD)	582 (95)	652 (115)	97.6 (2.02)	97.8 (1.67)

## Results

### Reaction times analyses

**Fig. 4:**
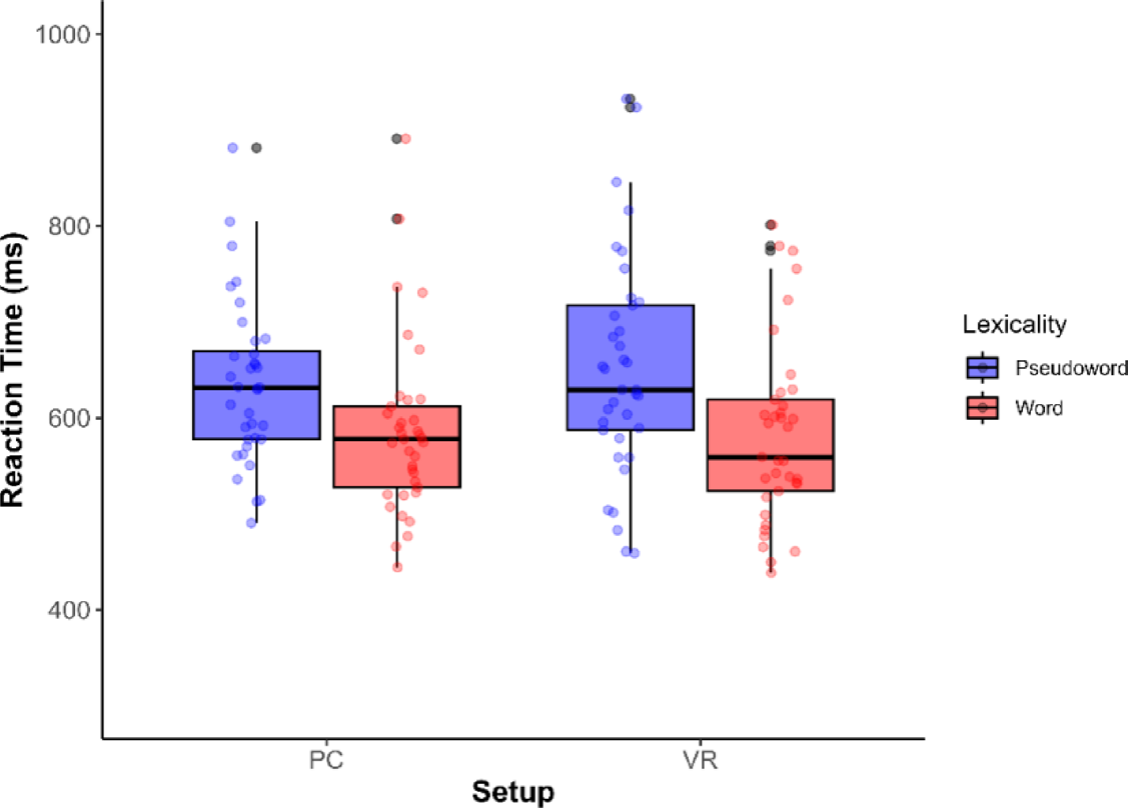
RT for each Setup and Lexicality levels.

ANOVA results are shown in Fig. 4. There was a significant main effect of Lexicality (F(1, 36) = 593.86, *p* < .001, η²ₚ = 0.623), with participants responding 67.2 ms faster to words than to pseudowords. No main effect of Setup (F[1, 36] = 0.04, *p* = .845, η²ₚ = 0.001), or interaction between Lexicality and Setup (F[1, 36] = 0.24, *p* = .628, η²ₚ = 0.007) was found. RT results are shown in Fig. 4.

Moreover, the supporting linear mixed-effect model showed an effect of Lexicality (b = -65.57, SE = 10.29, t = -6.37, *p* < .001), and no effect for Setup or interaction. Convergence issues necessitated simplification. The second model showed perfect correlations between random effects (*r* = 1), indicating over-specification. The final model confirmed robust Lexicality effects (words 68.15 ms faster than pseudowords, SE = 5.41, t(194.37) = -12.61, *p* < .001). Between-participant variability (SD = 99.00) and between-item variability (SD = 34.39) estimates were smaller than the residual trial-level variability (SD = 139.40). Diagnostic plots revealed approximately normal residuals despite a slight right skew in extreme values, with no evidence of heteroscedasticity.

### Accuracy analyses

**Fig. 5:**
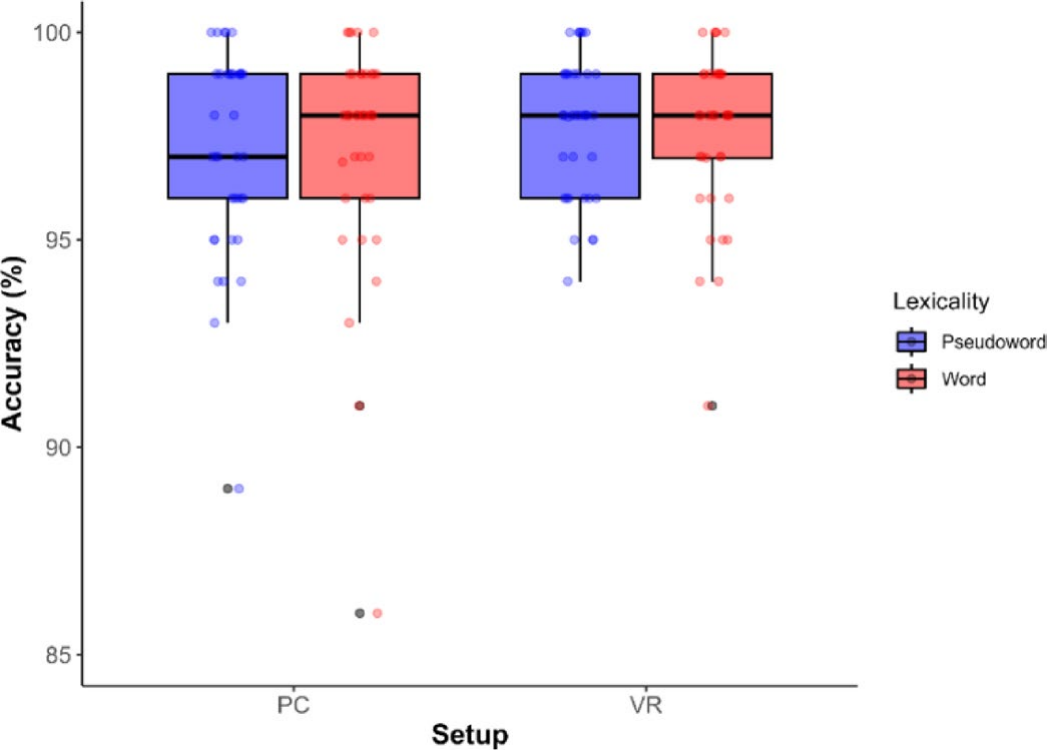
Accuracy percentages for each Setup and Lexicality levels.

The ANOVA analysis showed no significant main effect of Lexicality (F(1, 36) = 0.08, *p* = .778, η²*p* = .002), Setup (F(1, 36) = 2.83, *p* = .101, η²*p* = .073), nor interaction (F(1, 36) = 0.32, *p* = .574, η²*p* = .009). Accuracy results are shown in Fig. 5.

Regarding accuracy, the linear mixed-effects model revealed a significant effect of Setup (b = 0.209, SE = 0.100, z = 2.08, *p* = .037), with participants performing more accurately in the VR condition than in the PC condition. Post hoc comparisons confirmed this effect, showing that accuracy in the VR condition was significantly higher (difference = 0.811, SE = 0.0815, z = -2.08, *p* = .037). No significant effect of Lexicality was found (b = -0.134, SE = 0.159, z = -0.845, *p* = .398), nor was the interaction significant (b = -0.133, SE = 0.201, z = -0.664, *p* = .507). Diagnostic plots indicated that model assumptions were reasonably met.

## Discussion

In the present study, two analogous versions of the LDT were implemented in different setups, one on a VR HDM and the other on a PC monitor, under identical controlled conditions and using the same response collection devices. The stimuli were presented on a blackboard in a classroom, which provided a static but familiar environment in both setups. Results showed the lexicality effect, i.e., faster RTs for words compared to pseudowords. This effect remained stable across setups. Accuracy rates showed no significant effects of Lexicality and Setup. However, given the high Accuracy rates, this could also reflect a ceiling effect.

No differences were found between the ANOVA and LMM result patterns, indicating the robustness of ANOVA. In addition, the LMM analysis indicates that the majority of the variance in the data is due to variations within conditions and not to systematic participant or material-related differences. The consistent results across setups confirm the valid and reliable applicability of VR for the investigation of linguistic processes.

The present study aimed to isolate the reliability of stimuli presentation and behavioral responses in VR HMD systems establishing a foundational benchmark that opens a pathway to investigate other cognitive effects. The use of VR technology represents a logical step for immersive presentation and user interaction in scientific research of reading, as it supports innovative approaches to long-standing questions related to the way print is processed in real-life scenarios. One main advantage for that is its compatibility with eye-tracking, see Rocabado et al.^22^ for a recent example of reading in naturalistic VR environments with integrated eye-tracking. VR technology also allows for more fine-grained real-time data collection and analysis, using force-sensitive buttons as well as three-dimensional movement trajectory tracking (see^34^). Combining these with multimodal recordings, such as oculometry and electroencephalography (EEG), can provide a real-time behavioral correlate to physiological data, enhancing the capacity of research in this field^19,32,51^. The integration of stereophonic sounds, haptic devices (e.g., gloves; see^52^), and electrode-equipped suits are innovations that hold the potential to better simulate interaction with physical stimuli and offer a plethora of possibilities to explore the cognitive processes in the real world.

Notwithstanding the advantages of VR technology, concerns have been raised regarding the potential risks associated with its use. One such risk is cybersickness, which is characterized by episodes of nausea and a correlation with postural attitudes^53^ and may affect participant engagement and data quality^42,43,54^. Additionally, Mon-Williams & Wann^55^ posited that prolonged use of VR systems requiring maintained visual focus may culminate in long-term binocular vision impairments. However, more recent research has shown that using modern VR equipment for up to 40 min poses no risk to vision health^56–58^ and this is roughly the time of continuous recording involved in many language studies. The present study design shows stimuli in a fixed location, reducing avoids risks of cybersickness introduced by movement, navigation, or competing stimuli. Various tools have been developed to assess discomfort during VR engagement^59^ such as questionnaires and machine learning methods for predicting cybersickness^51^. However, such questionnaires have not yet been validated for Spanish-speaking populations, which limits the researcher’s ability to quantify and systematically monitor discomfort. Incorporating such measures in future experiments could lead to a better understanding of the potential risks associated with VR. Additionally, systems with built-in eye-tracking capabilities allow to control participant’s field of view, significantly reducing mobility-related distress (see^32^).

While the present study provides evidence supporting the use of VR for language research, certain limitations must be acknowledged. First, the sample covered a wide age range (18–59 years). To control for that, we used LMMs quantifying between-subject variability, which subsumes any potential age-related differences and showed limited effects. In addition, the sample consisted exclusively of male participants, which may limit the generalizability of our findings to the total population. Although not well established in this context, gender differences in lexical access or VR interaction could potentially influence outcomes.

Additionally, it is important to note that since our experiment utilizes an HMD VR setup, the generalization to other VR displays is limited. VR technology varies in display formats, with different degrees of immersion^60,61^ and interactivity^52,62,63^all of which might affect cognitive processing, attention, and user experience^42,43^. For example, desktop-based 3D applications and simple mobile VR systems, such as Google Cardboard, primarily offer limited passive experiences through 360° video, whereas PC-powered HMDs support interactive environments and enable real-time behavioral data collection (see^17,34^). Future research should explore whether different types of VR systems influence behavioral and cognitive outcomes, particularly in language research, where immersion and user agency may differentially affect processing.

In sum, the present study establishes VR as a reliable platform for investigating linguistic processes, demonstrating that well-established cognitive effects, commonly observed in traditional research settings, can be consistently replicated in VR. Although limited to an HMD setup and a male sample, these findings lay important groundwork for broader applications of VR in experimental cognitive research. As VR technology continues to evolve - incorporating tools for multimodal data integration, and enhanced sensory feedback - it holds significant promise for exploring language processing in ecologically valid contexts.

## Data Availability

The materials, data, and video demonstration of the experimental VR setting are available at the following OSF repository: https://osf.io/q3ec9/?view_only=c7924253f9fd419ba63d6cec635546ab.
